# Subtype-specific human endogenous retrovirus K102 envelope protein is a novel serum immunosuppressive biomarker of cancer

**DOI:** 10.3389/fimmu.2024.1533740

**Published:** 2025-01-09

**Authors:** Qinyuan Gong, Rongzhen Xu

**Affiliations:** ^1^ Department of Hematology and Cancer Institute (Key Laboratory of Cancer Prevention and Intervention, China National Ministry of Education), The Second Affiliated Hospital, Zhejiang University School of Medicine, Hangzhou, China; ^2^ Institute of Hematology, Zhejiang University, Hangzhou, China

**Keywords:** cancer immunosuppression, endogenous retroviruses, HML-2 subtype, HERV-K102, serum biomarker

## Abstract

Immune dysfunction is one of the hallmarks of cancer and plays critical roles in immunotherapy resistance, but there is no serum biomarker that can be used to evaluate immune-dysfunction status of cancer patients. Here, we identified subtype-specific human endogenous retrovirus K102 envelope (HERV-K102-Env) with immunosuppressive activity in circulating blood as a novel serum immunosuppressive biomarker of cancer. We first generated monoclonal antibodies against K102-Env with high sensitivity and specificity, and we developed an ELISA assay to detect serum K102-Env. We then investigated whether K102-Env and K108-Env proteins are present in circulating blood of cancer patients. We found K108-Env proteins were present in serum of both patients with cancer and healthy individuals. In contrast, K102-Env markedly increased in patients with PDAC, hepatocellular carcinoma (HCC), and non-small cell lung cancer (NSCLC) compared with healthy controls. The positive rates of K102-Env were 34.00%, 39%, and 28.0% in PDAC, HCC, and NSCLC, respectively, whereas only 5.0% of healthy individuals had marginally increased K102-Env. In the sera of PDAC patients, K102-Env was 36.63-fold higher than that of healthy controls. K102-Env significantly upregulated PD-1/PD-L1 and c-Myc expression levels of T cells. Importantly, serum K102-Env levels correlated well with advanced cancers and tumor biomarkers CA19-9 and AFP. These findings indicate that circulating K102-Env protein is a novel serum biomarker for evaluating immunosuppressive status and disease stage of patients with cancer.

## 1 Introduction

Immune cell dysfunction is a major barrier for cancer immunotherapies (1–6), but the exact causes remain incompletely defined, and there are still no serum biomarkers for evaluating the immune-dysfunction status of patients with cancer. Human endogenous retroviruses (HERVs) comprise 8% of the human genome (7, 8). Among HERVs, the HERV-K family is the most biologically active (9). Several HERV-K family members, such as HERV-K102 and HERV-K108, are unique to humans and are constantly evolving (10). We, and other researchers, have previously demonstrated that HERV-K envelope glycoproteins (K-Env), which consist of a surface unit (SU) and a transmembrane (TM) subunit with potential immunosuppressive activity, are enriched in tumor tissues and correlate with poor outcomes in cancers that include acute myeloid leukemia (AML), pancreatic duct adenocarcinoma (PDAC), lung cancer, and glioma (11–15). HERV-K promotes tumor development by inducing intercellular fusion in melanoma (16). Diseases such as ovarian cancer (17), prostate cancer (18), rheumatoid arthritis (19) and systemic lupus erythematosus (20) have also been associated with HERV-K activation. It is possible that the viral proteins encoded by HERV-K play an important role, but the exact oncogenic mechanism is not yet clear. Previous studies have shown that reactivation of HERV-K is associated with immunosuppressive status in some diseases. For example, HERV-K is persistently present in patients with AIDS who have failed to respond to HAART before and after HIV-1 rebounds, but it is undetectable in patients with successful HAART treatment (21). The TM subunit of HERV-K envelope has been shown to harbor potent immunosuppressive activity, and anti-HERV-K TM antibodies are present at a higher titer in HIV-1-infected adults who control HIV-1 in the absence of therapy (22). HERV-K is a supergroup of viruses, and there are at least 550 HERV-K proviruses integrated in the human genome (23). Of these, two proviruses, K102 and K108, contain an intact open reading frame for Env (24). However, it is unknown whether HERV-K Env protein is present in circulating blood of patients and which subtypes of HERV-K Env protein are specifically associated with cancer.

In this study, we first attempted to generate monoclonal antibodies against K102-Env by phage display library construction, and we developed an ELISA assay for serum K102-Env due to the lack of commercial ELISA assay kits. We then systematically assessed whether K102-Env and K108-Env proteins were present in the circulating blood of patients with cancer. Next, we investigated whether the circulating K-Env proteins in the blood were associated with disease activity and immune dysfunction of patients with cancer. Our results showed that subtype-specific K102-Env, but not K108-Env, was specifically present in the serum of patients with cancer. Moreover, K102-Env enhanced expression of PD-1 and c-Myc, two important regulators for promoting tumor immune evasion, in T cells (25–27). We further showed that circulating K102-Env levels correlated well with immunosuppressive status and disease activity of patients with cancer.

In recent years, the discovery and development of cancer biomarkers has experienced remarkable progress, and there are now widely applicable biomarker-based assays for some cancers, such as CA199 for pancreatic cancer and AFP for liver cancer. However, although some markers play an important role in diagnosis and prognosis, there are limitations to their application. For example, certain blood markers, such as carcinoembryonic antigen, have low diagnostic specificity and may lead to missed or misdiagnosis. Our study demonstrates for the first time that subtype-specific HERV-K102-Env is a novel immunosuppressive serum cancer biomarker that correlates with tumor markers and can be used to assess the state of immune dysfunction and the staging of cancer in cancer patients. This specific serum assay provides a novel approach to viral biomarker detection of cancer and even prediction of cancer metastasis.

## 2 Material and methods

Sera derived from patients with cancer and controls were collected using a standardized protocol and stored at −80°C until use. The age of the patients and healthy controls was not matched. All the samples were analyzed retrospectively, and the patients and controls were processed simultaneously. This study was performed after approval by the Ethics and Scientific Committee of The Second Affiliated Hospital of Zhejiang University School of Medicine.

### 2.1 Expression and purification of K102-Env-SU recombinant glycoprotein

To obtain high-quality monoclonal antibodies (mAbs) for detecting native HERV-K102-Env protein, we used K102-SU-hFc as the antigen. The K102-SU-hFc fusion gene was synthesized and cloned into the expression vector GSV0 with the hFc tag. The recombinant plasmid was transformed into Expi 293-T cells. Pierce™ Centrifuge Columns were used to achieve the purpose of purifying the Fc-tag fusion protein. The molecular weight and purity of K102 SU-hFc were evaluated by sodium dodecyl sulfate-polyacrylamide gel electrophoresis (SDS-PAGE).

### 2.2 Balb/c mouse immunization

Balb/c mice were immunized with 100 μg of K102-SU-hFc emulsified with an equal volume of Freund’s complete adjuvant through a subcutaneous route. After the first immunization, four additional injections were performed with 50 μg of protein emulsified with an equal volume of Freund’s incomplete adjuvant every 2 weeks. The sera were collected from the immunized mice, and the serum titers against K102-SU-hFc were analyzed by ELISA. Briefly, K102-SU-hFc (2 μg/mL) was coated at 4°C overnight and blocked with 5% PBSM (5% skim milk in PBS) at 37°C for 2 hours. Serially diluted sera were added and incubated at 37°C for 1 hour. Unimmunized serum was used as the negative control, and GN-mAb-Env-K01 was used as the positive control. Goat anti-mouse-IgG (1 + 2a+2b+3)-HRP and anti-human-Fc-HRP were used as the secondary antibodies.

### 2.3 Phage display library construction

Splenocytes were isolated from the immunized mice after the final immunization. Total RNA was extracted and used to synthesize cDNA by reverse transcription. PCR was employed for the amplification of the heavy chain and light chain of the gene of the antibody with two primer sets as described previously. Full-length genes were obtained by linking the light and heavy chain genes; the gene (approximately 1800 bp) was cloned into the pHEN2 phage display vector. After desalting and purification, the ligated products were converted by electrical transfer. After the transfer, 10 μL of the product was used for dilution assays and storage capacity calculation. A total of 2 μL of the product was used for sequencing. The remaining bacterial liquid was stored at −80°C with glycerin.

### 2.4 Screening and identification of antibodies against K102-Env-SU

We inoculated bacteria into 2YT (Car+Tet+) medium containing glucose and incubated it with shaking until OD600 ≈ 0.5, added Helper Phage for 1-1.5 hours, and incubated overnight to prepare phage suspension. The diluted antigen was added to the immunization tubes and incubated at 4°C overnight. The phage suspension was incubated with the protein-coated immunization tubes and then washed. A total of 800 µL of trypsin was added to the tubes, which were sealed and fixed on a rotator to elute at RT for 20 minutes. The eluate was mixed with *E. coli* SS320 cells and incubated at 37°C for 30 minutes, and then spread on 2YT-Car+Tet+ plates and incubated at 37°C overnight. Three rounds of screening were carried out, each using a three-fold gradient of decreasing antigen concentration (100 µg/mL, 30 µg/mL, 10 µg/mL), and the samples from the three rounds of screening were added to Helper Phage to obtain phage supernatant samples. The samples were diluted in a five-fold gradient, and the affinity test was performed by ELISA. Anti-M13-HRP antibody was used as the secondary antibody. After screening, a total of 534 clones were selected, 291 positive clones were obtained, 183 positive clones (OD > 1.68) were tested, and 19 molecules with unique sequences were obtained. After sequence-diversity analysis, 17 molecules were selected for full-length construction.

### 2.5 ELISA assay for serum K108-Env protein

For the examination of K108-Env protein in human sera, we employed a commercial Human HERV K Provirus Ancestral Env Polyprotein (ERVK6) ELISA Kit (CUSABIO, CSB-EL007812HU). The sera samples were collected and diluted 1:5 in sample dilution buffer. All the testing steps were processed according to the manufacturer’s instructions.

### 2.6 ELISA assay for serum K102-Env protein

Briefly, 96-well ELISA plates (Corning, 3690) were coated with mAb A115 (4 μg/mL) in PBS at 60 µL per well, and incubated at 4°C overnight. The next day, the plates were washed with PBST three times and blocked with 5% PBSM for 2 hours at RT. Then 1:5 diluted sera in 1% PBSM were added to the coated wells (60 μL/well) and incubated for 60 minutes at RT. K102-Env proteins at various concentrations were added to plot a standard curve. The plates were washed with PBST three times, and biotinylated mAb A164 (4 μg/mL) in 1% PBSM was added to the plates (60 µL/well) for 60 minutes at RT, followed by NeutrAvidin-HRP (Thermo, 31001) (1:2000 dilution) in PBS (60 µL/well) for 50 minutes at RT. The plates were developed by adding 70 μL of TMB substrate (Beyotime, P0209), followed by 70 μL of TMB stop solution (Beyotime, P0215). OD450 was measured on a Spectramax Absorbance Reader (Molecular Devices).

### 2.7 Construction of K102-Env or K102-Env-TM expression vectors

We transfected human Jurkat T cells using K102-Env or K102-Env-TM expression vectors. To produce lentiviruses, HEK293T cells in a 10-cm culture dish were transfected with a solution made of DMEM (500 µL, no FBS) together with 45 µL Polyjet transfection reagent (SignaGen, SL100688), 3 µg psPAX2 (Addgene, 12260; RRID: Addgene_12260), 3 µg pMD2.G (Addgene, 12259; RRID: Addgene_12259), and 9 µg lentiviral target plasmids (pcw-K102 Env-HA/pcw-K102 Env-Tm-HA). At 16 hours after transfection, supernatants from HEK293T cells were gently aspirated and replaced with pre‐warmed (37°C) culture medium. Viral supernatant was harvested at 48 and 72 hours after transfection, filtered through Amicon Ultra filters (Millipore, UFC910024), and added freshly to the cells along with 1 µg/mL Polybrene (Santa Cruz, sc‐134220). Transfections of plasmids were performed using Polyjet transfection reagent (SignaGen, SL100688) according to the manufacturer’s instructions. K102-Env or K102-Env-TM overexpression Jurkat T cells were obtained at 1–2 µg/mL puromycin (Sangon Biotech, A610593) selection.

### 2.8 Cell line and culture

Jurkat cells belong to the acute T-cell leukemia cell line. In this study, they were cultured in RPMI‐1640 medium supplemented with 10% FBS and 1% penicillin‐streptomycin. The cells were cultured in a humidified atmosphere with 5% CO_2_ at 37°C. The cell line was routinely tested for mycoplasma. Experiments were performed with noncontaminated cells.

### 2.9 Western blot analysis

Cells were collected and washed twice with PBS and then lysed on ice for 30 minutes in protein extraction reagent (Thermo Scientific, 78505) containing protease and phosphatase inhibitor (Thermo Scientific, 78443). The cell lysates were centrifuged at 13,000×g for 15 minutes. After the supernatant was collected, it was heated at 98°C for 5 minutes. The protein concentrations were assessed using a Pierce BCA Protein Assay Kit (Thermo Scientific, 23225). Equal amounts of protein samples were subjected to SDS‐PAGE and then transferred to PVDF membranes (Bio‐Rad), blocked with 5% nonfat milk in TBS‐T buffer, and then incubated with the indicated primary antibodies at 4°C overnight. Commercially valid antibodies were employed. All commercial antibodies were verified by the manufacturers using Western blots and/or images on their websites. Antibodies: GAPDH (60004‐1‐Ig), PD-1 (66220-1-Ig), PD‐L1 (66248‐1‐Ig) from Proteintech; c‐Myc (ab32072) from Abcam; HA (3724) from Cell Signaling Technology. After three washes with TBS‐T buffer, the membranes were probed with an HRP‐conjugated secondary antibody (goat anti-rabbit (HuaAn Biotechnology, HA1001) or goat anti-mouse (HuaAn Biotechnology, HA1006)) for 1 hour at RT, and signals were detected by a Tano 5200 Chemiluminescent Imaging System and quantified with ImageJ 1.52a software (https://imagej.nih.gov/ij/). All the experiments were repeated at least twice.

### 2.10 Statistical analysis

Parametric comparisons of normally distributed values that satisfied the variance criteria were made by unpaired Student’s t-test or one-way ANOVA with Bonferroni correction for multiple comparisons, data that did not pass the variance test were compared with Wilcoxon rank sum test. Differences with *p*‐value < 0.05 were considered statistically significant. Differences are labeled as follows: *p < 0.05; **p < 0.01; ***p < 0.001.

## 3 Results

### 3.1 Development and characterization of mAbs to K102-Env protein

**Figure 1:**
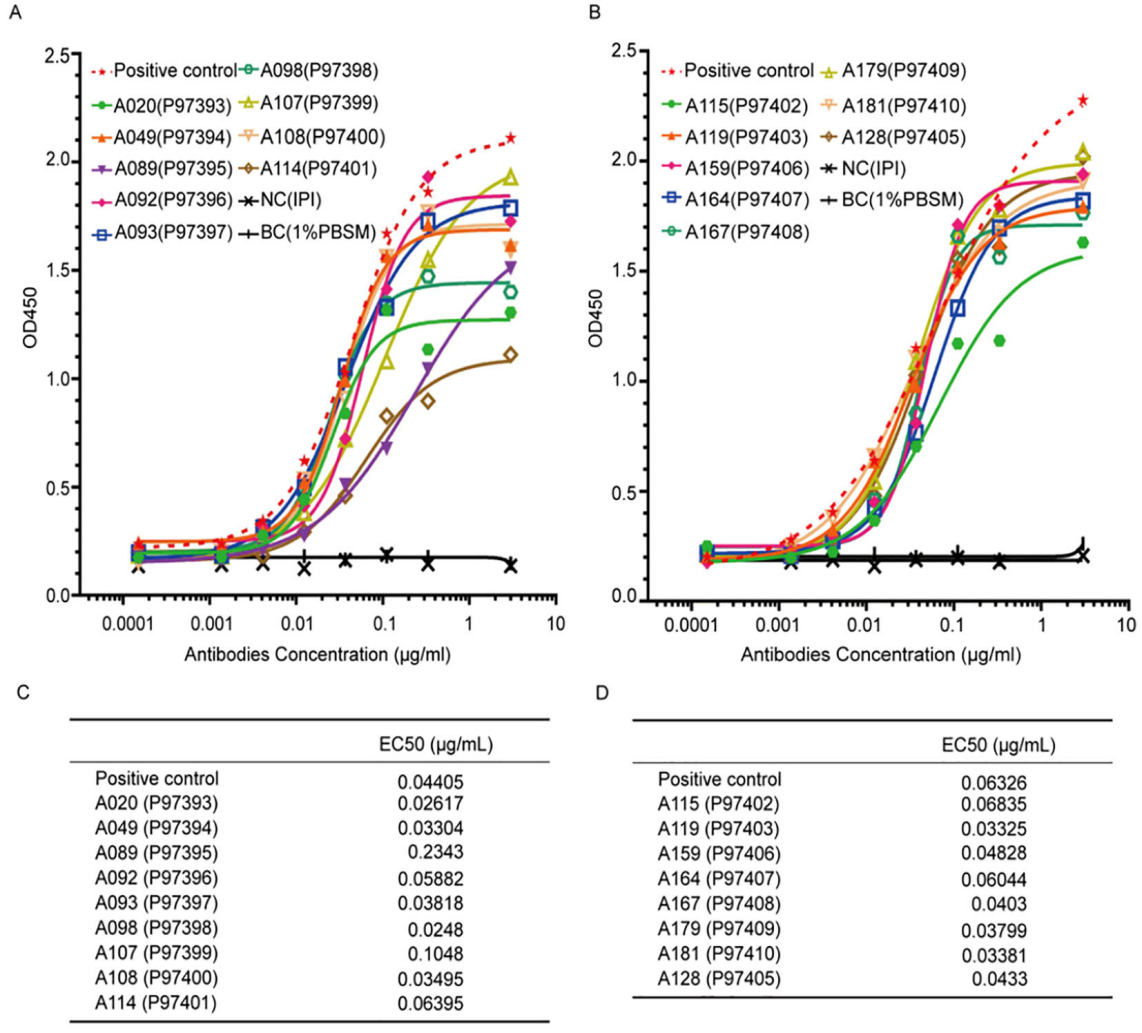
Specific binding assay and EC50 of 17 monoclonal antibodies (mAbs) against K102-Env-SU glycoprotein. **(A, B)** 17 mAbs against K102-Env-SU glycoprotein specifically bound to K102-Env-SU antigen in a dose-dependent manner measured by ELISA assay. **(C, D)** EC50 values of 17 mAbs against K102-Env-SU glycoprotein measured by ELISA assay.

Given the lack of a commercial ELISA kit for K102-Env, we first tried to develop monoclonal antibodies (mAbs) to the K102-Env protein. We used K102-Env-SU proteins to immunize BALB/c mice and then constructed a phage display library from the splenocytes of the immunized mice using standard techniques. After carrying out a high-throughput screening with K102-Env-SU antigens, a total of 17 mAb clones against K102-Env-SU glycoprotein were generated, with binding assay results and EC50 values are shown in **Figures 1A–D**. ELISA binding assay results revealed that mAb clones A115 and A164 had high specificity and sensitivity toward K102-Env-SU glycoprotein and exhibited the best pairing effect, and could be used to establish a double-antibody sandwich ELISA test for serum K102-Env proteins. mAb A115 was designated as the captured antibody, while biotin-coupled A164 served as the detector antibody in this assay. Taken together, we created a series of mAbs to K102 Env-SU with high specificity and sensitivity, which might be used to establish immunoassays for detecting K102 Env in sera and other biological samples.

### 3.2 Establishment of ELISA assay for HERV-K102-Env in serum

After obtaining a series of mAbs against K102-Env protein, we next attempted to develop a double-antibody sandwich ELISA quantitative assay kit for detecting subtype-specific HERV-K102 envelope with two specific mAbs, A115 and A164, against different epitopes of K102-Env-SU. First, 96-well ELISA plates were coated with mAb A115 as the captured antibody for 24 hours. The plates were washed with PBST and blocked with 5% PBSM. Then 1:5 diluted sera in 1% PBSM were added to the coated wells and incubated for 60 minutes at room temperature (RT). The plates were washed with PBST, and biotinylated mAb A164 as the detector antibody in 1% PBSM was added to the plates for 60 minutes at RT. NeutrAvidin-HRP in PBS was then added for 50 minutes at RT. The plates were developed by adding TMB substrate, followed by TMB stop solution. After measuring OD450 using a Spectramax Absorbance Reader (Molecular Devices) and adding K102-Env glycoproteins at various concentrations to create a standard curve. We found that the detection limit of this ELISA test was 0.03 ng/mL for detecting serum K102-Env.

### 3.3 K108-Env proteins are present in the serum of both patients with cancer and healthy individuals

**Figure 2:**
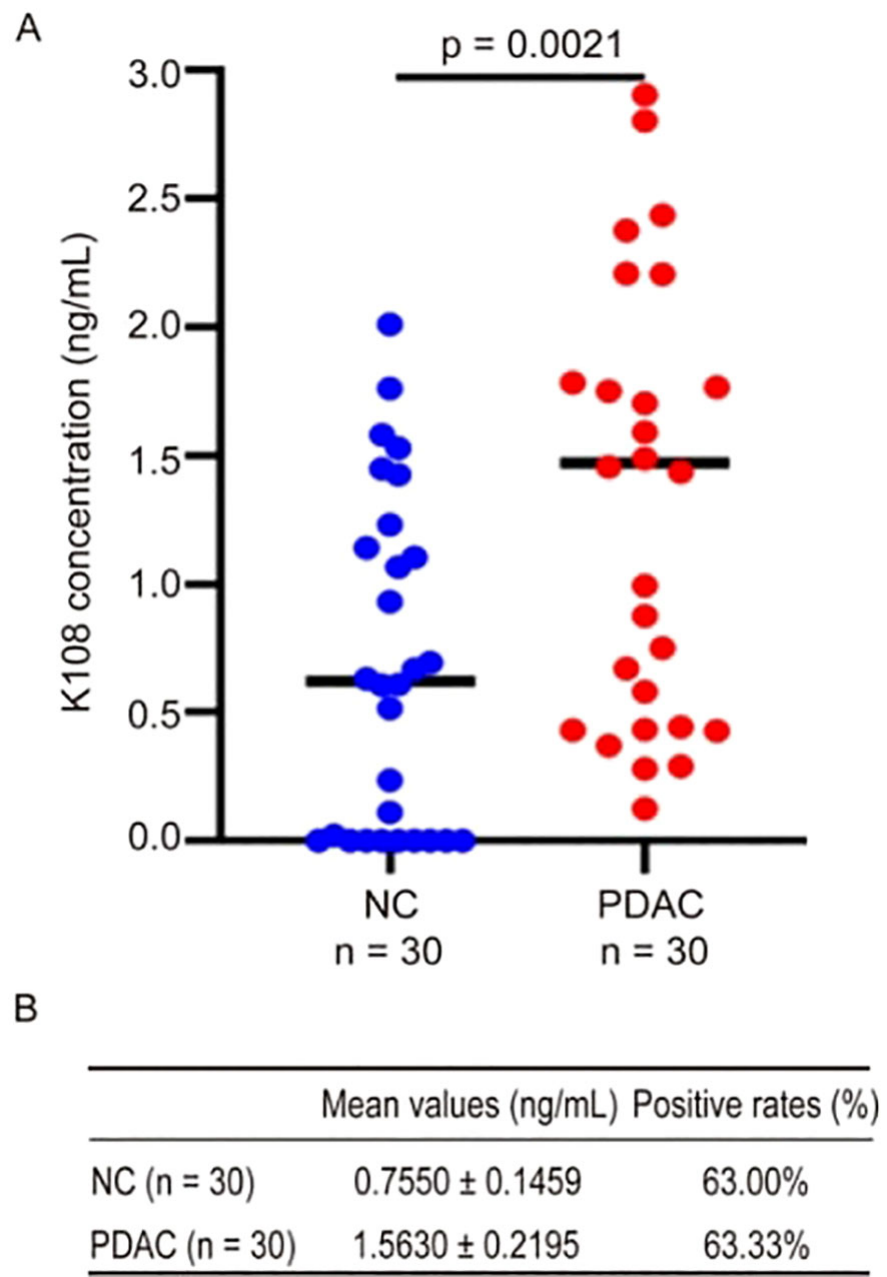
K108-Env proteins were detected in sera from patients with PDAC and healthy individuals. **(A)** Expression of K108-Env proteins in NC (n = 30) and PDAC (n = 30). **(B)** The mean values (mean ± S.D.) and positive rates of K108-Env protein in patients with cancer and healthy sera.

Previous studies have revealed that HERV-K-Env proteins are abundant in tumor tissues of a variety of cancers (11–15), and there are a number of HERV-K family members, but it is uncertain if K-Env proteins are present in the circulating blood of patients with cancer. Of the HERV-K family, only HERV-K108 and HERV-K102 proviruses contain intact ORFs (24), thus, we focused on K108-Env and K102-Env in this study. For the detection of K108-Env in human sera, we used a commercial ELISA kit for K108-Env with a detection limit of 0.15 ng/mL. ELISA was performed in accordance with the manufacturer’s instructions. We detected K108-Env proteins in both PDAC and healthy sera. K108-Env proteins were found in 63.33% of PDAC patients (n = 30) and in 63% of healthy sera (n = 30) (**Figures 2A, B**). The mean values of K108-Env in patients with cancer and healthy sera were 1.56 ng/mL and 0.76 ng/mL, respectively (**Figure 2B**). Interestingly, while K108-Env proteins were expressed in sera of healthy individuals, K108-Env expression levels were significantly upregulated in patients with PDAC. These results suggest that K108-Env proteins exist in circulating blood but are not tumor-specific.

### 3.4 K102-Env proteins are specifically present in the serum of patients with cancer

**Figure 3:**
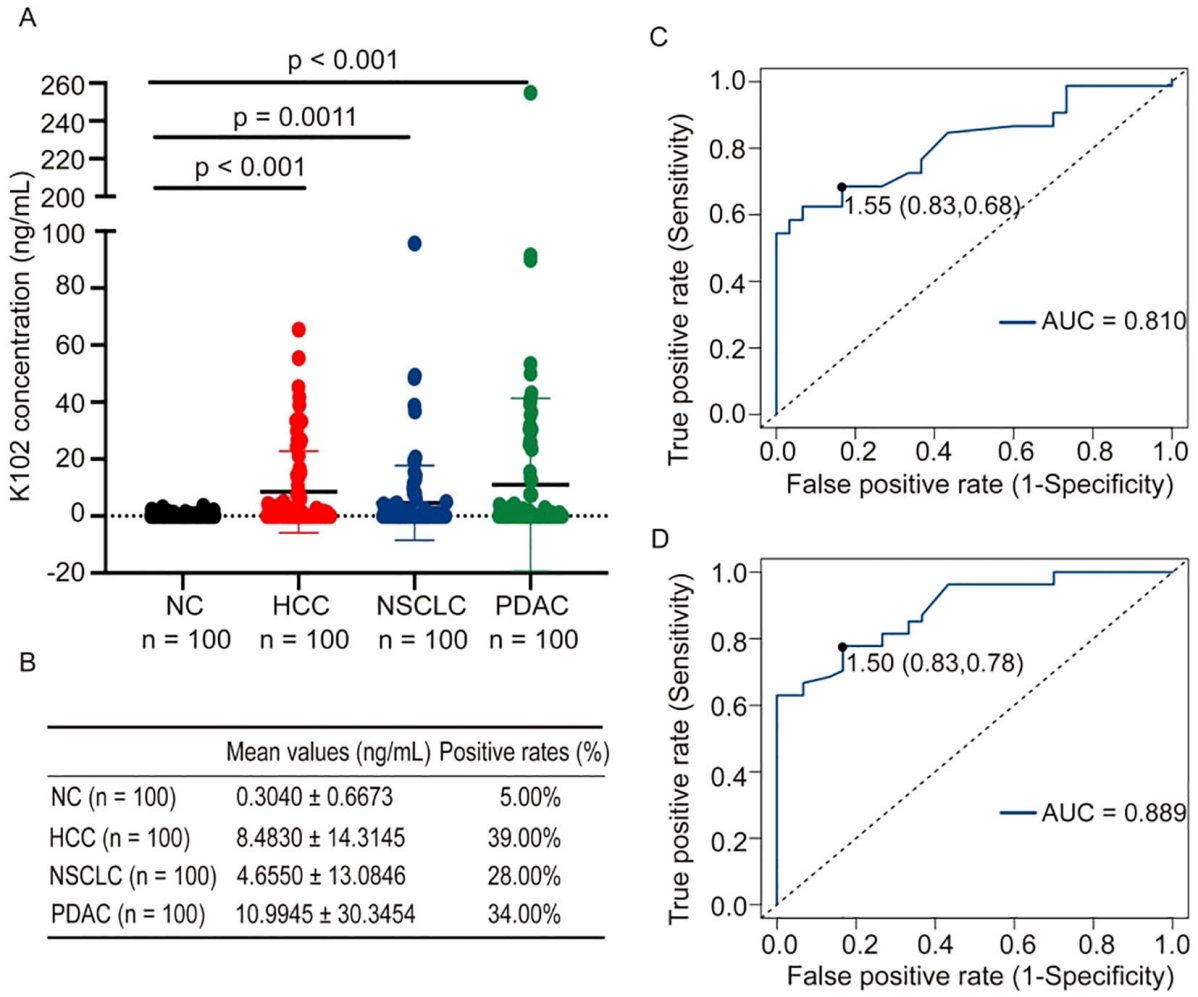
K102-Env proteins were detected in sera from patients with cancer and healthy individuals. **(A)** Expression of K102-Env proteins in NC (n = 100), and patients with HCC (n = 100), NSCLC (n = 100) and PDAC (n = 100). K102-Env was significantly increased in patients with HCC (p < 0.001), NSCLC (p = 0.0011) and PDAC (p < 0.001) compared to healthy controls. **(B)** The mean values (mean ± S.D.) and positive rates of K108-Env protein in patients with cancer and healthy sera. **(C)** ROC curve of K102-Env proteins in patients with PDAC. **(D)** ROC curve of K102-Env proteins in patients with HCC.

We have shown that K108-Env proteins are present in the sera of both patients with cancer and healthy individuals. To reveal which subtypes of HERV-K-Env proteins are specifically present in the sera of patients with cancer, we next measured K102-Env protein levels in the sera of patients with cancer and healthy individuals. In contrast to K108-Env proteins, which were commonly present in both sera of patients with cancer and healthy individuals, K102-Env was considerably higher in patients with PDAC, HCC and NSCLC than in healthy controls (p < 0.01) (**Figure 3A**). K102-Env was detected in 34.00% of patients with PDAC (n = 100), 39% of patients with HCC (n = 100), and 28.0% of patients with NSCLC (n = 100), whereas only 5.0% of healthy individuals (n = 100) had marginally increased HERV-K102 (**Figure 3B**). The mean values of K102-Env in the sera of patients with PDAC, HCC, and NSCLC and healthy controls were 10.99, 8.48, 4.66, and 0.30 ng/mL, respectively (**Figure 3B**). ROC curves were used to assess the positive rates of K102-Env protein levels. For the PDAC curve, the area under the ROC curve (AUC) was 0.81, and the optimum cutoff value was 1.55 ng/mL, at which we achieved sensitivity of 68% and specificity of 83% in all patients with PDAC (**Figure 3C**). For the HCC curve, the AUC was 0.889, and the optimum cutoff value was 1.50 ng/mL, at which we achieved sensitivity of 78% and specificity of 83% in patients with HCC (**Figure 3D**). Moreover, K102-Env protein levels are much higher than those of K108-Env in patients with cancer. The K102-Env protein level in patients with PDAC was 36.63-fold higher than that in healthy controls. In addition, 5.0% of healthy individuals also showed low levels of K102 Env proteins. These results indicate that subtype-specific K102-Env is a tumor-associated immunosuppressive protein in the serum of patients with cancer.

### 3.5 Serum K102-Env levels correlate with known tumor markers

**Figure 4:**
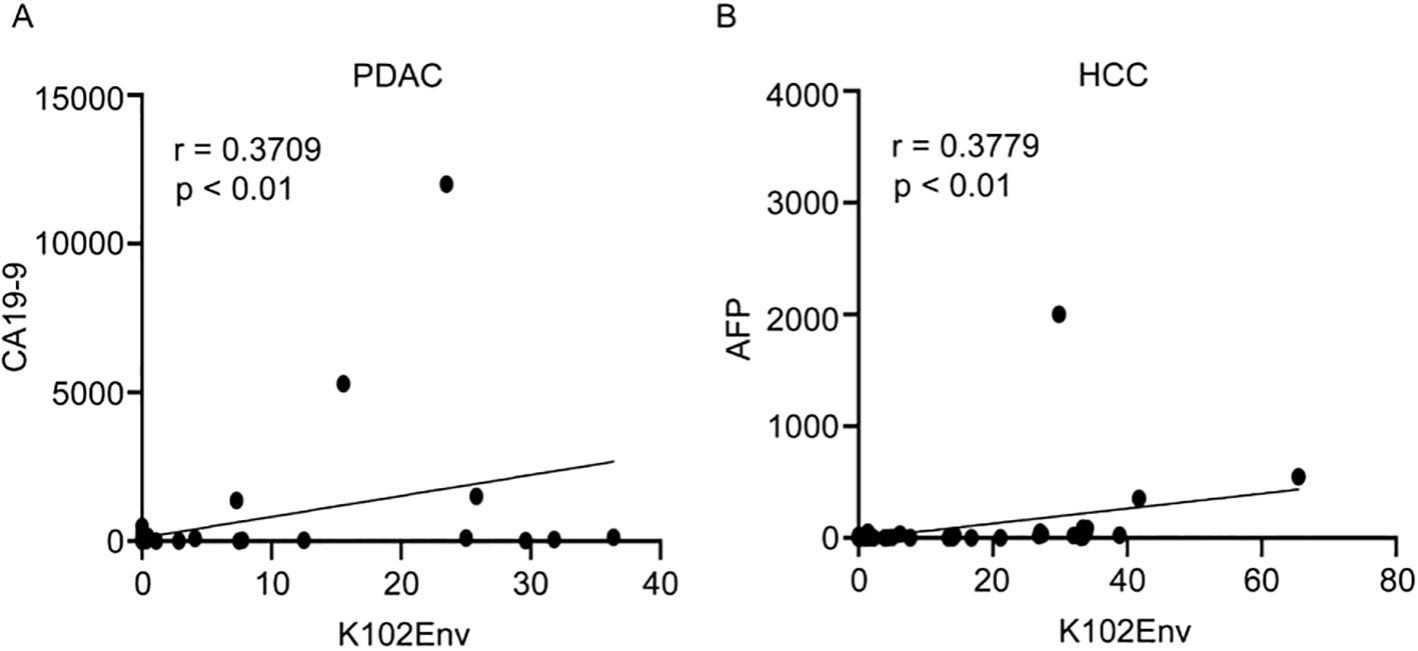
Serum K102-Env levels correlate with known tumor markers. **(A)** K102-Env levels and CA19-9 levels in 54 patients with PDAC were analyzed with a positive correlation (p < 0.01). **(B)** K102-Env levels and AFP levels in 65 patients with HCC were analyzed with a positive correlation (p < 0.01).

Given that carbohydrate antigen 19-9 (CA19-9) and alpha-fetoprotein (AFP) are two well-known serum biomarkers for PDAC and HCC, respectively, we next evaluated whether circulating K102-Env levels correlate with these biomarkers in patients with PDAC and HCC. We conducted an analysis of K102-Env levels and CA19-9 levels in 54 patients with PDAC, and concluded that there is a strong correlation between K102-Env and CA19-9 levels (p < 0.01) (**Figure 4A**). Similarly, we found a positive correlation between K102-Env and AFP in 65 patients with HCC (p < 0.01) (**Figure 4B**). These results further support that K102-Env is a tumor-associated serum biomarker.

### 3.6 Serum K102 Env levels are associated with advanced cancers

**Figure 5:**
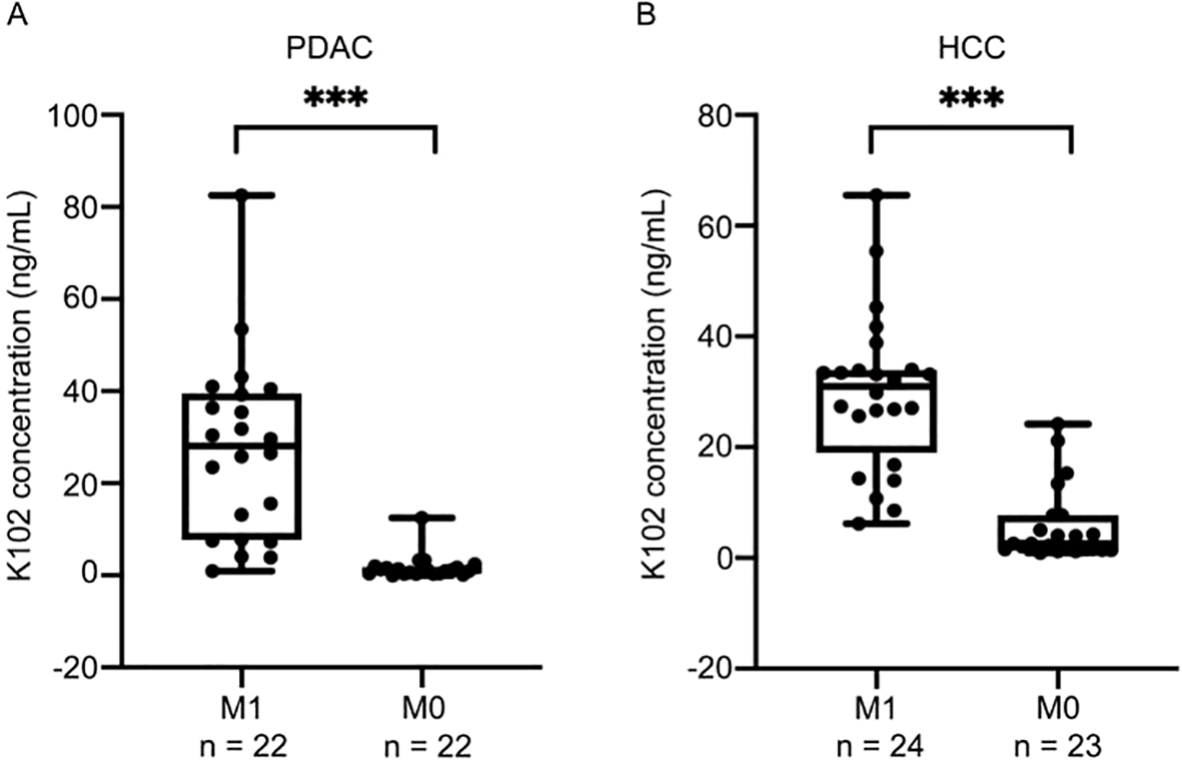
Serum K102-Env levels are associated with advanced cancers. **(A)** boxplots depicting K102-Env levels for 22 patients with distant metastases (M1) and 22 patients without distant metastases (M0) in PDAC. The difference is significant (p < 0.001). **(B)** boxplots depicting K102-Env levels for 24 patients with distant metastases (M1) and 23 patients without distant metastases (M0) in HCC. The difference is significant. (***p < 0.001).

While high levels of HERV-K-Env in tumor tissues imply poor outcome, it is unknown whether elevated circulating K102-Env in blood is associated with disease activity. We next analyzed the correlation of circulating K102-Env levels and cancer stage in PDAC and HCC, and we found increased circulating K102-Env proteins mainly in the sera from patients with advanced or metastatic cancer. We compared 22 patients with distant metastases (M1) and 22 patients without distant metastases (M0) in PDAC (**Figure 5A**), and 24 patients with distant metastases (M1) and 23 patients without distant metastases (M0) in HCC (**Figure 5B**), and we found that the patients who developed metastatic disease had higher levels of serum K102-Env proteins. This difference was significant (p < 0.001) (**Figures 5A, B**). These results suggest that circulating K102-Env could be used as a biomarker to evaluate cancer stage.

### 3.7 K102-Env upregulates PD-1/PD-L1 and c-Myc of T cells

**Figure 6:**
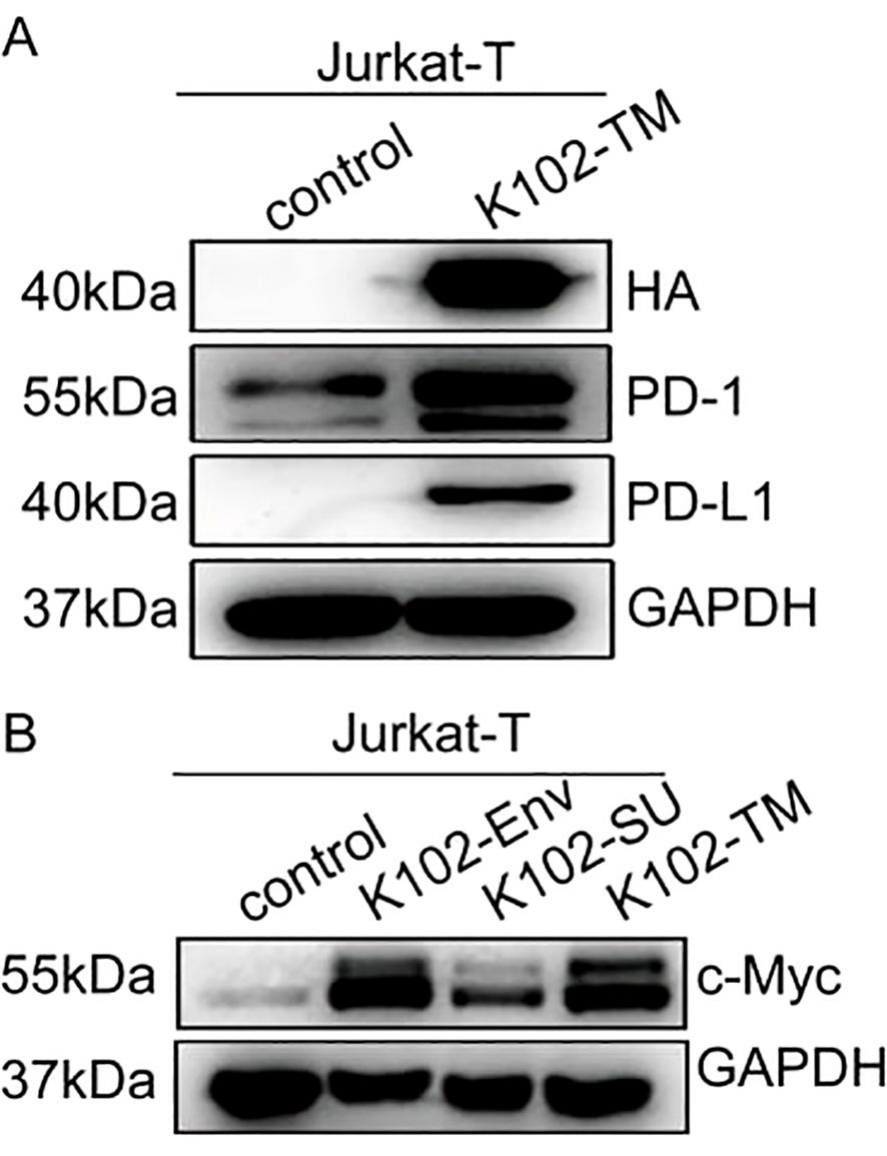
K102 Env upregulates PD-1/PD-L1 and c-Myc of T cells. **(A)** Western blot analysis for PD-1 and PD-L1 in Jurkat-T cells transfected by the K102 Env-TM-HA expression vector. **(B)** Western blot analysis for c-Myc in Jurkat-T cells transfected by K102-Env-HA or K102 Env-TM-HA expression vectors.

PD-1 and PD-L1 are two critical drivers of immune evasion of tumors, and MYC has been shown to suppress antitumor immune response by upregulating the PD-L1 (28, 29). However, it is unknown whether K102-Env regulates these cellular checkpoints and oncogenes. We next examined whether K102-Env expression activates c-Myc, PD-1 and PD-L1 in T cells. We transfected human Jurkat-T cells using K102-Env or K102-Env-TM expression vectors, and we found that overexpression of K102-Env or K102-Env-TM led to a marked upregulation of c-Myc, PD-1 and PD-L1 (**Figures 6A, B**). These findings indicate that K102 Env might play a critical role in cellular checkpoint-mediated antitumor immune evasion, and increased circulating K102-Env may contribute to the immune dysfunction of cancer.

## 4 Discussion

We and other researchers have shown that HERV-K envelope glycoproteins harbor immunosuppressive properties and are enriched in tumor tissues of a variety of cancers, which are associated with poor outcomes. In the present study, we, for the first time, demonstrated that subtype-specific K102-Env, but not K108-Env, is a novel serum biomarker for evaluating immunosuppressive status and disease stage of patients with cancer. Serum antibodies against HERV-K-Env proteins have been reported to be present in some patients with cancer, such as lung cancer (14), breast cancer (30), and prostate cancer (31), but the antibody titer is generally low or absent in most patients with advanced cancer (14). Thus, the HERV-K-Env antibody is not a reliable indicator for evaluating the immune function status of patients with advanced cancer. HERV-K envelope glycoproteins harbor immunosuppressive activity and could inhibit the function of various immune cells (32), but it is unknown whether cancer-specific HERV-K-Env proteins are present in the circulating blood of patients, and whether circulating K-Env is associated with immune dysfunction. To address these questions, we systematically investigated the circulating K108-Env and K102-Env proteins in the blood of patients with different types of cancers and healthy controls, and their relationship with immune dysfunction as well as cancer stage.

Since no commercial ELISA kit for K102 Env is available, we first generated a series of mAbs against K102-Env-SU protein via phage display library construction and a high-throughput screening with HERV-K102-SU antigens. We acquired a total of 17 mAb clones against K102-Env-SU protein with high sensitivity and specificity. Among the 17 mAb clones, A115 and A164 exhibited the best pairing effect and were used to establish a double-antibody sandwich ELISA assay with a detection limit of 0.03 ng/mL for serum K102-Env. The mAb A115 was designated as the captured antibody, and biotin-coupled A164 served as the detector antibody in this assay. By using this simple and accurate ELISA assay, it is possible to screen K102 Env proteins in serum and other fluids on a large scale.

An important finding of this study is that subtype-specific K102-Env, but not K108, is abundantly and specifically present in the circulating blood of patients with cancer. We compared the protein levels of K102-Env and K108-Env in the circulating blood of patients with a variety of types of cancers and healthy individuals with ELISA assays. We found that K102-Env was present in 34.00% of patients with PDAC, 39% of patients with HCC, and 28.0% of patients with NSCLC, whereas only 5.0% of healthy individuals had marginally increased HERV-K102. Importantly, we demonstrated that circulating K102-Env levels were closely linked to tumor biomarkers CA19-9 and AFP, as well as with advanced cancer in patients with PDAC and HCC. In contrast, we found that K108-Env was present in the sera of both patients with PDAC and healthy individuals, but K108-Env levels were significantly higher in patients with cancer. In patients with PDAC who have a high resistance to immunotherapy, circulating levels of K102-Env protein were 36.63-fold higher than those in the sera of healthy controls. These findings indicate that increased circulating K102-Env protein is a novel serum tumor-associated biomarker and might predict disease stages of patients with cancer.

Of note, our study results show that K102-Env upregulates expression of cellular checkpoints PD-1/PD-L1 and oncogene c-Myc in T cells. It has been reported that endogenous retroviruses activate the cyclic GMP-AMP synthase (cGAS)/stimulator of interferon genes protein (STING) signaling pathway, which affects interferon secretion (33). Activated IFN-α enhances the induction and maintenance of PD-1 expression on T cells involved in T-cell receptor (TCR) through the association of IFN response factor 9 (IRF9) with IFN-stimulated regulatory elements (ISRE) (34). Studies have shown that physical and functional interactions of human endogenous retrovirus proteins Np9 and Rec with the promyelocytic leukemia zinc finger protein (PLZF). One major target of PLZF is the c-Myc proto-oncogene. Coexpression of Np9 and Rec with PLZF abrogates the transcriptional repression of the c-Myc gene promoter by PLZF and results in c-Myc overproduction (35). Based on the above findings, the specific molecular mechanisms by which K102-Env leads to the upregulation of PD-1, and c-Myc in T cells need to be further explored. PD-1 and PD-L1 are two critical regulators of immune evasion of tumors, and c-Myc has been shown to suppress antitumor immune response by upregulating the PD-L1 (28, 29). These findings indicate that circulating K102-Env is a serum immunosuppressive biomarker of patients with cancer, and it could be used to evaluate the immune-dysfunction status of patients with cancer, especially those patients with advanced cancer.

Finally, the discovery of circulating K102-Env in the blood suggests that the circulating K102-Env with immunosuppressive activity may affect the antitumor function of immune cells in tissues and organs with circulating blood. Clearly, this new hypothesis needs further investigation. There are some limitations to this study, our sample size is not large enough, the study is limited to a few cancer types, and an in-depth *in vivo* study of the biological function of HERV-K102-Env is lacking. In the follow-up study, we intend to increase the sample size of collected patients and expand the study scope to more cancer types. At the same time, we will further investigate the specific pathogenic mechanism of HERV-K102-Env in cancer and confirm it with a series of *in vivo* and ex vivo experiments. Our work is the first to show that subtype-specific HERV-K102-Env, but not K108-Env, is a novel serum cancer-associated biomarker for evaluating immune-dysfunction status and disease activity of patients with cancer.

## Data Availability

The original contributions presented in the study are included in the article/supplementary material. Further inquiries can be directed to the corresponding author.
