# Corrigendum: Subtype-specific human endogenous retrovirus K102 envelope protein is a novel serum immunosuppressive biomarker of cancer

**DOI:** 10.3389/fimmu.2025.1560546

**Published:** 2025-01-30

**Authors:** Qinyuan Gong, Rongzhen Xu

**Affiliations:** ^1^ Department of Hematology and Cancer Institute (Key Laboratory of Cancer Prevention and Intervention, China National Ministry of Education), The Second Affiliated Hospital, Zhejiang University School of Medicine, Hangzhou, China; ^2^ Institute of Hematology, Zhejiang University, Hangzhou, China

**Keywords:** cancer immunosuppression, endogenous retroviruses, HML-2 subtype, HERV-K102, serum biomarker

In the published article, there was an error in the correspondence section:

Instead of “*CORRESPONDENCE Rongzhen Xu email: zrxyk2024@163.com”, it should be “*CORRESPONDENCE Rongzhen Xu email: zrxyk10@zju.edu.cn”.

The authors apologize for this error and state that this does not change the scientific conclusions of the article in any way. The original article has been updated.

